# Breast Cancer Stem Cells Survive Periods of Farnesyl-Transferase Inhibitor-Induced Dormancy by Undergoing Autophagy

**DOI:** 10.1155/2011/362938

**Published:** 2011-07-07

**Authors:** Moumita Chaterjee, Kenneth L. van Golen

**Affiliations:** ^1^The Laboratory of Cytoskeletal Physiology, Department of Biological Sciences, University of Delaware, Newark, DE 19716, USA; ^2^The Center For Translational Cancer Research, University of Delaware, 320 Wolf Hall, Newark, DE 19716, USA

## Abstract

A cancer stem cell has been defined as a cell within a tumor that possesses the capacity to self-renew and to cause the heterogeneous lineages of cancer cells that comprise the tumor. These tumor-forming cells could hypothetically originate from stem, progenitor, or differentiated cells. Previously, we have shown that breast cancer cells with low metastatic potential can be induced into a reversible state of dormancy by farnesyl transferase inhibitors (FTIs). Dormancy was induced by changes in RhoA and RhoC GTPases. Specifically, RhoA was found to be hypoactivated while RhoC was hyperactivated. In the current study we demonstrate that these dormant cells also express certain known stem cell markers such as aldehyde dehydrogenase I (ALDHI) and cluster of differentiation 44 (CD44). We also show that autophagy markers Atg5, Atg12, and LC3-B are expressed in these dormant stem cell-like breast cancer cells. Inhibiting autophagy by inhibitor 3-methyladenine (3-MA) blocked the process of autophagy reversing the dormant phenotype. Further, we show that c-jun NH2 terminal kinase (JNK/SAPK) is upregulated in these dormant stem cell-like breast cancer cells and is responsible for increasing autophagy.

## 1. Introduction

Metastatic breast cancer is the leading cause of cancer death in women in the United States [1]. Improvements in the understanding of the molecular underpinnings of breast cancer, coupled with improvements in multimodality treatments have greatly improved the overall survival of breast cancer patients [1–3]. However, a subset of women who appear to have been “cured” of their cancer recur years, if not decades, later [4]. These recurrences are due to the presence of latent or dormant tumor cells [5–7]. Often dormant tumor cells, termed disseminated tumor cells, can be detected in the bone marrow of women in remission of their cancer [8, 9]. 

The mechanisms of tumor cell dormancy are currently not understood. Our laboratory has demonstrated that treatment of breast tumor cells with farnesyl transferase inhibitors (FTIs) leads to a phenotype reminiscent of dormancy [10, 11]. Further, FTI treatment of the MCF-7 cell line leads to profound changes in Rho GTPase activation [10]. Specifically, RhoA GTPase becomes hypoactivated while RhoC GTPase becomes hyperactivated. These changes lead to radical changes in the cell cytoskeleton and cellular morphology. Decreased levels of RhoA activation are also consistent with an accepted *in vitro* model of breast cancer cell dormancy [12–14]. 

Similar to what is observed for the *in vitro* model [12, 13], FTI-induced dormancy is reversible. Upon FTI withdrawal cells grow normally after exiting from nearly two weeks of dormancy [10]. FTI-treated cells appear to have minimal metabolic activity, yet remain viable. Thus, we believe that under these conditions the cells have undergone the process of autophagy. Autophagy is the process where a cell degrades organelles such as mitochondria to expend less energy avoiding apoptosis [15]. Autophagy is regulated through the extracellular matrix and is suggested to be required for dormancy [16–18]. In addition, activation of the c-jun NH2 terminal kinase (JNK/SAPK) signaling pathway occurs during autophagy [19, 20]. In our previous study we demonstrated that increased RhoC GTPase activation during FTI treatment increased JNK/SAPK signaling leading to breast tumor cell dormancy [10]. 

It has been suggested that breast cancer cells with stem cell-like properties are responsible for metastatic spread [21]. These cells express stem cell markers such as aldehyde dehydrogenase I (ALDHI) and cluster of differentiation 44 (CD44) as well as RhoC GTPase [22–24]. Furthermore, it is suggested that dormant breast cancer cells are of cancer stem cell origin [21, 25]. The stem cell origin of dormant cells would help explain how outgrowth of the dormant cell can lead to rapid tumor growth and dissemination. Recent reports suggest that the MCF-7 cell line is comprised of a large proportion of cells displaying stem cell-like properties [26, 27]. MCF-7 cells readily undergo dormancy in different *in vitro *and *in vivo* models including FTI-treatment [10]. 

In the current study we hypothesized that the breast tumor cells affected by FTI treatment would express breast tumor stem cell markers. Further, we hypothesized that FTI-induced changes in Rho GTPase activation leading to increased JNK/SAPK signaling would induce autophagy in these cells. Here we show that cells affected by FTI treatment express breast cancer stem cell markers ALDH1 and CD44. In addition, these cells appear to be undergoing autophagy through a RhoC GTPase- and JNK/SAPK-dependent signaling pathway. These data are the first to demonstrate the role of stem cells and autophagy in FTI-induced breast tumor cell dormancy and may have implication for future therapeutic uses of FTIs.

## 2. Materials and Methods

### 2.1. Cell Culture, FTI Treatment, and Transfections

MCF-7 breast cancer cell line was obtained from American Type Culture Collection (Manassas, VA) and maintained at 37°C with 5% CO_2_ levels in DMEM (Mediatech, VA)/10% FBS (Altanta Biologicals, GA)/1% Penicillin-Streptomycin (Mediatech, VA). Cell line was validated for authenticity by the Johns Hopkins Genetics Resource Core Facility. FTI L-744,832 was kindly provided by Dr. George Prendergast (Lankenau Institute for Medical Research) and treatment for dormancy was performed as previously described [10]. 

Transfections were performed using constitutively active and dominant negative mutant clones of RhoC, G14V, and T19N, respectively (Missouri S&T cDNA Resource Center). FugeneHD (Roche) was used as the transfection reagent and transfection performed according to the manufacturers instructions.

For cells treated with the JNK inhibitor SP600125 (EMD Biosciences, Gibbstown, NJ): MCF-7 cells were plated at a clonogenic density of 10,000 cells per well in six-well plates and treated with 25 *μ*M of FTI for 24 h. 25 *μ*M SP600125, pharmacological inhibitor of JNK, was added in each well in 2 mL of medium along with 0.25% sterile dimethyl sulfoxide (DMSO). As control, 0.25% DMSO in 2 mL of medium was added to the FTI-alone treated cells. 3-Methyladenine (3-MA) (Calbiochem, CA) was dissolved in dimethylformamide (DMF) and added to cells originally plated at a clonogenic density of 10,000 cells per well in 6-well plates after dormancy was induced by treating them with 25 *μ*M FTI for 24 h. The working concentration of 3-MA was 5 mM.

### 2.2. Immunofluorescence

Dormancy was induced for 72 h by FTI treatment, the cells fixed with 4% paraformaldehyde, extracted in 0.1% Triton-X-100 in PBS, blocked with blocking buffer containing 3% Bovine Serum Albumin (BSA) plus 10% normal rabbit or goat serum, respectively. Cells were then incubated with either anti-ALDH1 mouse primary antibody (1 : 1000) (BD Transduction Labs) or anti-CD44 rabbit primary antibody (1 : 1000) (Strategic Diagnostics, Newark, DE). For ALDH1 staining, Alexa Fluor 568 rabbit anti-mouse secondary antibody (Molecular Probes, Invitrogen) was used while Alexa Fluor 488 goat anti-rabbit secondary antibody (Molecular Probes, Invitrogen) was used for CD44 staining. Draq5 (Biostatus Limited, UK) was used to stain the nuclei in both cases. IgG controls were used in both cases as negative controls. Immunofluorescence was performed on a Ziess LSM5 High-speed Live confocal microscope housed in Delaware Biotechnology Institute.

### 2.3. Western Blotting

Western blotting was performed as previously described [10]. Briefly, proteins were harvested using RIPA buffer containing protease inhibitor cocktail. Lysates were separated by SDS-PAGE on a 4–20% (w/v) gel (Biorad, Hercules, CA) transferred to nitrocellulose, blocked and probed with a monoclonal antibodies for phosphorylated and total JNK (Cell Signaling, Beverly, MA) and autophagy marker LC3B and other genes using the autophagy sampler kit (Cell Signaling, MA). After incubation with a goat anti-rabbit-horseradish peroxidase (HRP) (Cell Signaling, MA) immunoblots were developed with ECL, exposed to Hyperfilm (Amersham, Piscataway, NJ) and images recorded on an Alpha Image 90 documentation system (Alpha Innotech, San Leandro, CA).

### 2.4. TUNEL Assay

TUNEL staining was performed using the APO-BrdU TUNEL Assay kit from Molecular Probes (Invitrogen, Eugene, OR). Briefly, cells were washed and fixed in 1% paraformaldehyde and let stand overnight in ice-cold ethanol at −20°C. After two repeats of pelleting, aspirating supernatant, and washing, cells were incubated in a DNA-labeling solution for 60 minutes at 37°C. At the end of the incubation time, cells were rinsed and pelleted twice after which they were incubated with the antibody staining solution containing Alexa Fluor 488 dye-labeled anti-BrdU antibody for 30 min and then deposited on slides and imaged using the LSM 5-LIVE High Speed Confocal housed in Delaware Biotechnology Institute, Newark, DE.

### 2.5. Statistical Analysis

All experiments were performed a minimum of three separate times with individual transfections or treatments and performed with no less than three replicates per experiment. Statistical analysis of the combined experiments was performed using GraphPad Prism. Significance was defined as a *P* value ≤0.001. Data is represented as mean ± standard deviation.

## 3. Results

### 3.1. FTI-Responsive MCF-7 Cells Express Stem Cell Markers

**Figure 1:**
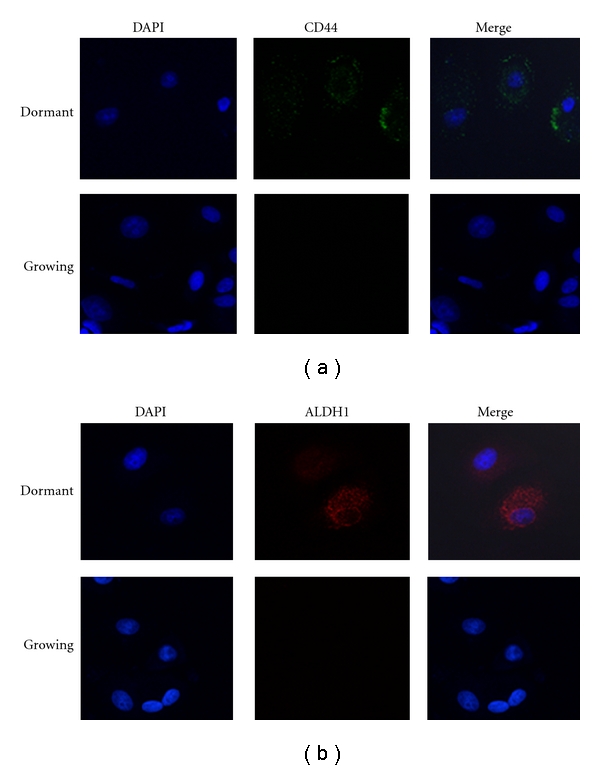
Immunofluorescence of markers associated with breast cancer stem cells. MCF-7 were treated with 25 *μ*M FTI for 72 h, fixed with paraformaldehyde, and incubated with antibodies specific for CD44 or ALDH1. Shown is a comparison of dormant and growing MCF-7 cells.

Previously, we demonstrated that a significant number of MCF-7 cells entered into a dormant phenotype when treated with 25 *μ*M of the farnesyl transferase inhibitor (FTI) L-744,832 [10]. To determine whether the dormant breast cancer cells expressed known breast cancer stem cell markers we performed immunofluorescence for aldehyde dehydrogenase 1 (ALDH1) and cluster of differentiation 44 (CD44) on FTI-treated MCF-7 cells (Figure 1). As described previously, FTI treatment induced a flattened and spread morphology associated with dormancy in a large number of MCF-7 cells. Cells that displayed the dormant morphology stained positive for ALDH1 and CD44. In contrast, growing MCF-7 cells did not stain for these described breast cancer stem cell markers. These data suggest that MCF-7 cells susceptible to FTI-induced dormancy may have stem-like properties.

### 3.2. FTI-Responsive Cells Express Early Markers of Autophagy

**Figure 2:**
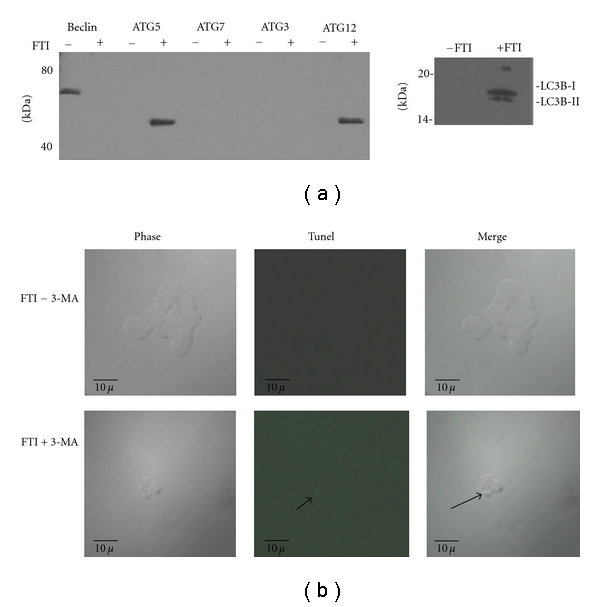
Expression of autophagy markers in FTI-treated cells. (a) Subconfluent MCF-7 cells were treated with 25 *μ*M FTI or vehicle control for 24 h, cell lysates harvested and western blot analysis performed with antibodies for autophagy-associated proteins. (b) MCF-7 cells were treated with FTI for 48 h and then 5 mM 3-MA or vehicle control added to the cultures. Cells were fixed 24 h later and a TUNEL assay performed. The arrow represents a single positive apoptotic cell.

Cells treated with FTI can remain in a dormant state for nearly two weeks and maintain viability [10, 11]. It has been suggested that dormant cancer cells undergo autophagy to remain viable [16, 18]. Next, we queried whether FTI treatment induced autophagy in these cells to promote cell survival under normal growth conditions.  Figure 2(a) is western blot analysis for the autophagy markers beclin, Atg5, Atg7, Atg3, Atg12, and LC3B expressed by vehicle control and FTI-treated MCF-7 cells. After 24 h FTI treatment, cells began to display early changes in the protein expression of autophagy markers indicative of autophagy. Interestingly, we observed a loss of beclin expression when the cells were treated with FTI. We did not detect Atg7 or Atg3 in untreated or after 24 h FTI treatment. However, detectable levels of the Atg12 and Atg5 conjugate were observed in the FTI-treated cells. LC3B-I is typically expressed in cells and its cleavage to LC3B-II is indicative of autophagy. In our cells we did not observe expression of LC3B-I or -II in growing (-FTI) MCF-7 cells. Expression of LC3B-I and LC3B-II was detected when the cells were treated with FTI. 

We next used a pharmacologic inhibitor of autophagy; 3-methyladenine (3-MA) is a specific inhibitor of the autophagic/lysosomal pathway.  Figure 2(b) is a comparison of FTI-treated MCF-7 cells with and without the addition of 3-MA. Co-treatment of 3-MA and FTI led to a significant decrease in cells displaying a dormant phenotype. As we previously demonstrated, dormant cells are significantly larger and spread out compared to nondormant, growing cells [10]. We observed that the 3-MA treated cells reverted from a large, spread morphology to a normal morphology. Further, we expected that inhibition of autophagy with 3-MA would significantly increase apoptosis in the FTI-treated cells. Using a TUNEL assay we found that very few cells were undergoing apoptosis upon 3-MA treatment. Together, these data suggest that induction of autophagy helps maintain the FTI-induced dormant phenotype but that inhibition of the autophagic/lysosomal pathway does not lead to increased apoptosis, at least in the early stages of autophagy.

### 3.3. RhoC GTPase Is Required for FTI-Induced Dormancy

**Figure 3:**
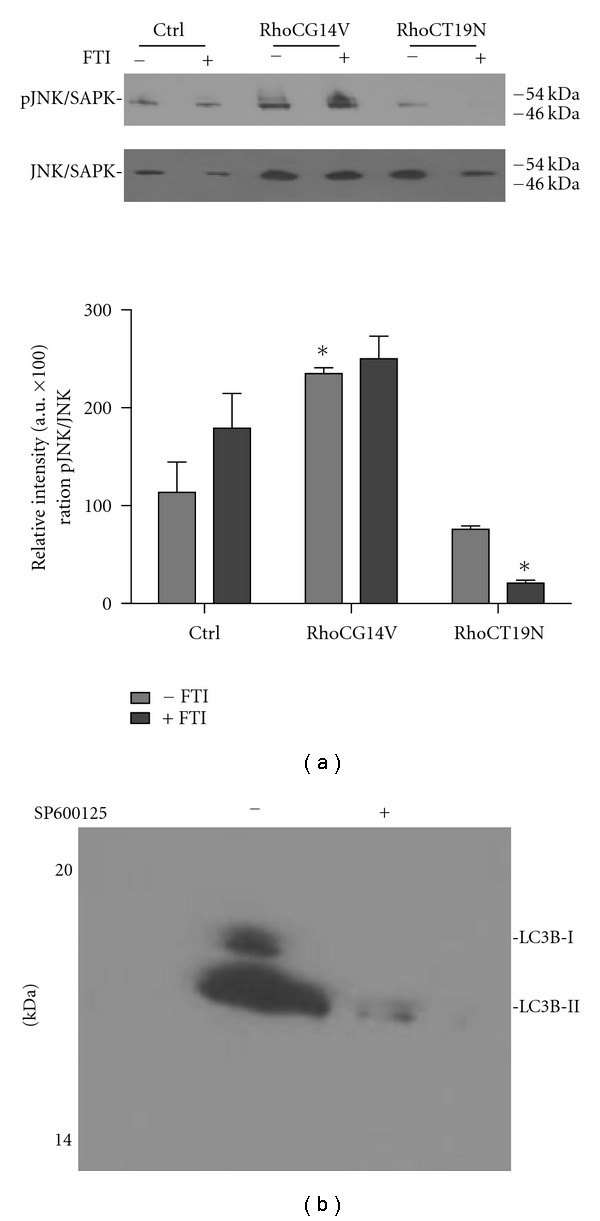
Effect of RhoC GTPase on JNK/SAPK activation. (a) MCF-7 cells were transiently transfected with empty vector (ctrl), a dominant active (G14V), or dominant negative (T19N) RhoC GTPase, treated with 25 *μ*M FTI or vehicle control and levels of active and total JNK/SAPK measured by western blot analysis. Shown is a representative western blot. Densitometry was performed using ImageJ and results of the ratio of active: total JNK represented as arbitrary units (AU). For significance *P ≤ 0.001. (b) representative western blot analysis of LC3B-I/-II levels in FTI-treated cells after inhibition of JNK/SAPK with 25 *μ*M SP600125.

RhoC GTPase expression and activation is suggested to influence the breast cancer stem cell phenotype [22, 28]. Additionally, we have demonstrated that RhoC hyperactivation drives breast cancer cell dormancy, potentially through JNK/SAPK [10]. To determine if RhoC activation drives the dormant phenotype by induction of JNK/SAPK signaling and autophagy, we modulated RhoC activation in control and FTI-treated cells. MCF-7 cells were transfected with either a dominant active (G14V) or negative (T19N) RhoC GTPase, treated with FTI and the levels of active JNK/SAPK determined.  Figure 3(a) is western blot analysis of phosphorylated and total JNK/SAPK. Control cells were transfected with empty vector. In comparison with the control cells, active JNK/SAPK levels in RhoCG14V were increased regardless of FTI treatment. A significant difference was particularly noted when the vehicle control treated RhoCG14V transfected cells were compared to the vehicle-treated empty vector control cells. In contrast, expression of a dominant negative RhoC led to a significant decrease in phospho-JNK/SAPK levels in vehicle and FTI-treated cells. Significance was determined between the vector control and RhoCT19N transfected cells treated with FTI. These data suggest that RhoC activity affects JNK/SAPK activation through RhoC GTPase activation. Further, RhoC activation via FTI-treatment leads to increased JNK/SAPK activity.

Next, we set out to determine if JNK/SAPK activity regulates FTI-induced autophagy in MCF-7 cells. Cells were pretreated with the JNK/SAPK inhibitor SP600125 and then incubated with FTI.  Figure 3(b) is western blot analysis demonstrating that FTI-stimulated JNK/SAPK activation is inhibited with SP600125 pretreatment. Next, we assessed expression of the autophagy marker LC3B JNK/SAPK inhibition. SP600125 inhibition of FTI-induced JNK/SAPK signaling prevented LC3B-I/-II expression in FTI-treated MCF-7 cells, suggesting JNK/SAPK activation leads to induction of autophagy.

## 4. Discussion

A great deal of hope and effort has been put into the development of farnesyl transferase inhibitors, however they have proven to be clinically disappointing. Attention has been focused on the Rho GTPases as potential targets for FTIs [29–34]. Evidence from our laboratory and others suggested that inflammatory breast cancer (IBC) patients may benefit from FTI treatment [11] and reviewed in [35]. The unique invasive IBC phenotype is due to expression of high levels of RhoC GTPase [36–38]. The RhoC-driven invasive IBC phenotype is inhibited by FTI treatment [11]. However, we found that these FTI-treated IBC cells were resistant to cell death and morphologically resembled what has been described for an *in vitro* model of breast cancer cell dormancy. Recently, we demonstrated that cells with low metastatic potential were susceptible to FTI-induced dormancy [10]. 

Tumor cell dormancy is a major clinical concern. Years or even decades after a breast cancer patient is deemed “cured”, highly aggressive recurrences can arise [21]. Very little is known about the molecular basis of dormancy. It is known that single cells can lie dormant in bone marrow. Alternatively, small groups of cells lacking a proper blood supply can lie dormant in the parenchyma of visceral organs. The mechanisms of what keep these cells dormant or what releases them are currently unknown and are an area of focus. 

Markers such as ALDH1 and CD44 are shown to be expressed by a subpopulation of cells in both tumors and cells lines [23, 24, 39]. It is suggested that breast cancer cells with stem cell properties are responsible for metastatic spread [21, 40]. Further, RhoC GTPase is suggested to be expressed by highly metastatic cells that exhibit “stemness” [22, 28]. Cancer stem cells have also been linked to dormancy. It is thought that a metastatic stem cell arriving in a nonconducive environment undergoes prolonged dormancy [21]. This, in part, would explain why recurrences from dormant cells can be so aggressive. 

In the current study we demonstrate that cells undergoing dormancy after FTI treatment, express ALDH1 and CD44. In contrast to breast cancer cell lines with greater metastatic capabilities such as MDA-MB-231, the majority of MCF-7 cells are susceptible to FTI-treatment and become dormant [10]. This may be because the MCF-7 breast cancer cell line has a large population of cells which have stem cell-like properties and express ALDH1 and CD44 [26, 27].

Dormant cells are also thought to undergo autophagy in order to survive [16–18]. Here we demonstrate that FTI-treated cells express early markers of autophagy as compared to controls. Pharmacologic inhibition of the autophagic/lysosomal pathway leads to a reversion of the FTI-induced dormant phenotype. Interestingly, it does not lead to apoptosis of the FTI-treated cells. Evidence from Liu et al. suggests an additional stress such as serum deprivation or inhibition of Akt1 may be needed to induce apoptosis in FTI-treated MCF-7 cells [33, 41]. 

Induction of autophagy is linked to the JNK/SAPK pathway [20, 42, 43]. Previously, we demonstrated that FTI-treated cells exhibited drastic alterations in RhoA and RhoC GTPase activation [10]. Specifically, RhoA became hypoactivated while RhoC became hyperactivated. Hyperactivation of RhoC was tied to increased JNK/SAPK activation and dormancy. We demonstrate that JNK/SAPK activation is increased when a dominant active RhoC is introduced into the cells. The levels of JNK/SAPK activation are similar to control cells treated with FTI and can be abrogated by expression of a dominant negative RhoC. Direct inhibition of the JNK/SAPK pathway also leads to reversion of the dormant phenotype (data not shown) and inhibition of the autophagic marker LC3B-I/-II.

Together these data suggest that cells expressing the stem cell markers ALDH1 and CD44 are susceptible to FTI-induced dormancy. This phenotype is due to JNK/SAPK signaling resulting from increased RhoC activation. In turn JNK/SAPK signaling leads to induction of autophagy allowing the cells to remain inactive. These data may have implications for the use of FTIs in the clinic, limiting to tumors that have a particular gene profile. Potentially, FTI-induced dormancy could synchronize tumor cells; FTI withdrawal would allow growth making the cells more susceptible to chemotherapeutics. In addition, this study may shed light on the mechanisms of breast cancer dormancy.
